# Portland Cement Use in Dental Root Perforations: A Long Term Followup

**DOI:** 10.1155/2014/637693

**Published:** 2014-03-03

**Authors:** Álvaro Henrique Borges, Matheus Coelho Bandeca, Mateus Rodrigues Tonetto, Luis Augusto Faitaroni, Elibel Reginna de Siqueira Carvalho, Juliane Maria Guerreiro-Tanomaru, Mário Tanomaru Filho

**Affiliations:** ^1^Faculty of Dentistry, University of Cuiabá, Brazil; ^2^Faculty of Dentistry, State University of São Paulo, Rua Humaitá no. 1680, Centro, 14801-903 Araraquara, SP, Brazil

## Abstract

Root canal and furcal perforations are causes of endodontic therapy failure and different materials that stimulate tissue mineralization have been proposed for perforation treatment. In the first case, a patient presented tooth 46 with unsatisfactory endodontic treatment and a periapical radiographic lesion. A radiolucent area compatible with a perforating internal resorption cavity was found in the mesial root. The granulation tissue was removed, and root canals were prepared. The intracanal medication was composed of calcium hydroxide and the perforation cavity was filled with Portland cement. The 11-year followup showed radiographic repair of the tissue adjacent to the perforation and absence of clinical signs and symptoms or periapical lesion. In the second case, a patient presented with edema on the buccal surface of tooth 46. The examination showed a radiolucent area in the furcation region compatible with an iatrogenic perforation cavity. The mesial root canals were calcified, and only the distal root canal was prepared. The cavity was filled with a calcium hydroxide-based paste and the distal root canal was obturated. In sequence, the perforation cavity was filled with Portland cement. The 9-year followup showed the tooth in masticatory function with radiographic and clinical aspects compatible with normality.

## 1. Introduction

In different stages of endodontic treatment, accidents may occur due to the complexity of the internal dental anatomy and inadequate planning. The most frequent accidents during endodontic treatment are root canal deviation (i.e., apical step and transportation), fracture of endodontic instrument, and root canal perforations [1, 2]. Root canal perforations are defined as the communication between the pulp cavity and the periodontal tissue and alveolar bone. Perforations have iatrogenic or pathological aetiologies that involve caries or resorption [3]. They may occur on the pulp-chamber floor during root canal location and prosthetic space preparations for radicular post [1]. These accidents are the second largest cause of failures and represent approximately 10% of unsuccessful endodontic treatments [4, 5]. Several materials, such as zinc oxide and eugenol, glass ionomer cements, and composite resins, have been suggested to repair root canal perforations [5, 6].

In 1993, Torabinejad developed Mineral Trioxide Aggregate (MTA) at the Loma Linda University [7]. In 1999, MTA was approved for human use by the Food and Drug Administration (FDA) and commercially is available as ProRoot MTA (Tulsa Dental, Oklahoma, USA) and MTA Ângelus (Ângelus, Soluções Odontológicas Ltda, Londrina-PR, Brazil) [8]. MTA is currently the most indicated material for root-end fillings and repair of root canal perforations [9]. This material has excellent physical [10], chemical [10, 11], and biological properties [12]. It is considered a biomaterial, and its ability to induce mineralized tissue may be related to the presence of calcium phosphate [13].

MTA consists of Portland cement (PC) that is associated with bismuth oxide, being used as a radiopacifier [14, 15]. Several studies have used PC as a substitute for MTA [16–19]. PC shows antimicrobial activity [15] and presents biocompatibility similar to MTA and low genotoxicity [6]. When being placed in contact with pulp tissue, MTA and PC provide the same tissue response for direct pulp capping [17] and pulpotomy [19, 20].

The clinical application of PC in humans has been reported. For periapical repair, PC has been used as an apical plug on an immature tooth [16]. Clinical and radiographic success (after one year) was reported using PC in pulpotomies of mandibular primary molars in children [18]. The formation of mineralised tissue was observed in 100% of 5- to 9-year-old children with mandibular primary molars that were treated with PC, after six months of treatment [19].

Due to their similar compositions, PC has long been considered to be a possible substitute for MTA in endodontic applications [20, 21]. The present report describes the long followup of two cases using Portland cement to repair root canal perforations.

## 2. Report 

### 2.1. Case 1

**Figure 1:**
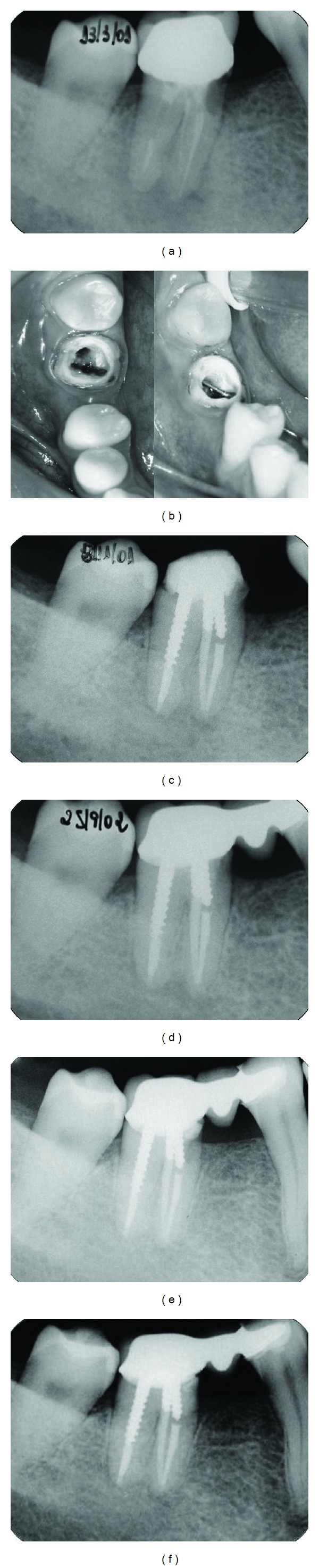
(a) The radiographic evaluation of case 1. (b) Clinical evaluation of the case. (c) Radiographic evaluation after perforation sealing 10 months later, 3 years later (d), 6 years later (e), and 11 years later (f).

A 37-year-old female patient in good general health sought treatment at the School of Dentistry from the Cuiabá University, Brazil. A periapical radiographic examination showed unsatisfactory endodontic treatment and asymptomatic apical periodontitis in tooth 46 (Figure 1(a)). A radiolucent area that was compatible with a perforating internal resorption cavity was found in the mesiolingual surface of the mesial root. A clinical examination showed a probing depth that was consistent with the presence of a periodontal pocket. Considering the root resorption and the periodontal bone loss, the extraction of the dental element and placement of an implant were proposed. The possibility of maintaining the tooth through endodontic retreatment and the subsequent treatment of the root canal perforation with a biological sealing material was presented as an alternative.

The maintenance of the dental element was the treatment of choice. Thus, the prosthetic crown was removed, and the remaining tooth was evaluated (Figure 1(b)). The granulation tissue in the resorption area was removed using periodontal curettes, and the bleeding was controlled with irrigation and a 1% sodium hypochlorite solution (Biodinâmica, Quím. e Farm., Paraná, Brazil). The endodontic retreatment and the removal of the filling material were performed with K-files (Dentsply Maillefer, Ballaigues, Switzerland) and an orange oil solvent. Gates-Glidden burs (number 1 and number 2) were used in the cervical and middle thirds (Dentsply Maillefer, Ballaigues, Switzerland). The mesial root canal was prepared up to a K-40 file, and the distal root canal was prepared up to a K-50 file, until the working length. The root canals were irrigated at each instrument change with a 1% sodium hypochlorite solution that was alternated with 17% ethylenediaminetetraacetic acid (EDTA) (Odahcam; Dentsply, Petrópolis, RJ, Brazil). Following the preparation, the root canals were filled with a calcium hydroxide paste (Biodinâmica, Quím. e Farm., Paraná, Brazil) and saline solution. After four monthly changes of intracanal medication, the root canals were filled with Sealapex sealer (SybronEndo, Sybron Dental Specialties, USA) and gutta-percha cones. The perforation cavity was filled with grey Portland cement (Itaú-Votorantin, Mato Grosso, Brazil) that was manipulated with distilled water. Clinical and radiographic monitoring was performed at ten months, and the prosthesis was prepared (Figure 1(c)). The monitoring of the restored and functional tooth was performed at 3 (Figure 1(d)), 6 (Figure 1(e)), and 11 years (Figure 1(f)). The monitoring revealed the absence of pain, fistulas, edema, and periodontal pockets, as well as a normal tissue colour and radiographic repair.

### 2.2. Case 2

**Figure 2:**
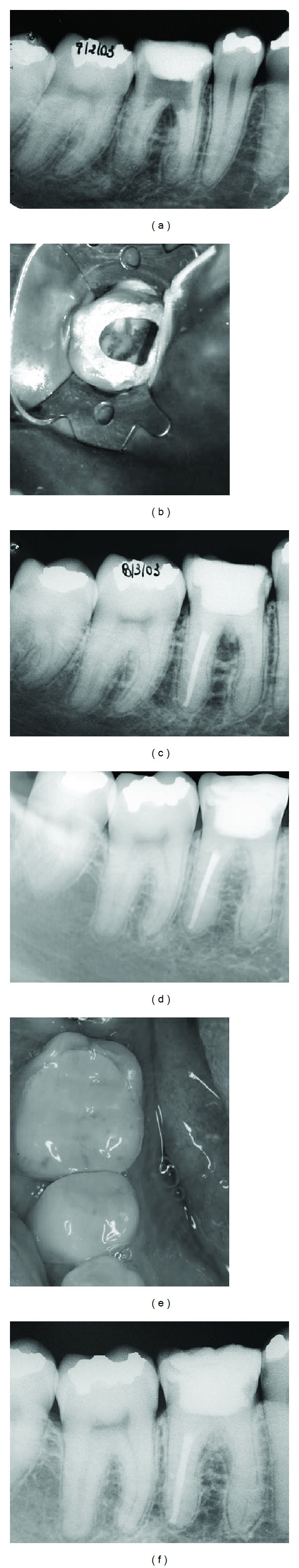
(a) Radiographic evaluation after perforation treatment. (b) Clinical aspect after the cavity filling. (c) Radiograph control 6 months later, 4 years later (d). (e) Clinical view after the tooth restoration and radiograph control 9 years later (f).

A 35-year-old female patient in good general health was referred to the Cuiabá University, Brazil, for endodontic treatment due to a dental 4 pain. The clinical examination showed edema on the buccal surface of tooth 46. The crown opening had been performed before the appointment. The radiographic examination showed a radiolucent area in the furcation region, which was compatible with a bur perforation (Figure 2(a)). The granulation tissue was removed and irrigation using 1% sodium hypochlorite was performed.

The mesial root canals were not found during the endodontic treatment due to their calcification. The cervical and middle thirds of distal root canal were prepared with Gates-Glidden burs (number 1 and number 2), and root canal was prepared up to a K-50 file at the working length. The root canal irrigation was performed at each instrument change using 1% sodium hypochlorite and 17% EDTA. After the preparation, the root canal and perforation cavity were filled for 30 days with calcium hydroxide paste. The distal canal was filled with Sealapex sealer and gutta-percha cones. The perforation cavity was sealed using grey Portland cement mixed with distilled water (Figure 2(b)). A glass ionomer cement was placed over the Portland cement. In the final radiograph, Portland cement extravasation was noted in the perforation cavity area. Clinical (Figure 2(e)) and radiographic monitoring was performed at six months (Figure 2(c)), four years (Figure 2(d)), and nine years (Figure 2(f)). Absence of pain, fistulas, edema, and periodontal pockets, as well as a normal tissue colour and radiographic repair were observed.

## 3. Discussion

Root and furcal perforations represent a leading cause of endodontic therapy failure [3]. Proper treatment can be performed by the two different ways: access through the radicular root canal or by surgical access to the external root surface [4, 22]. The location of the root perforation, time between perforation and treatment, presence of contamination, and physicochemical and biological properties of the used sealing material determine the success of the treatment [3, 22].

These two case reports showed the occurrence of perforations involving different strategies of conservative treatment. To achieve success in the treatment of perforations, a correct treatment planning involving several specialties is important. In the first case, the perforating cavity was diagnosed in the cervical region of tooth 46. This perforation may have been related to a process of caries or an iatrogenic procedure or root canal resorption. The cause of the perforation in the second case was an iatrogenic procedure related to an error while performing the location of mesial root canals in tooth 36.

In cases of perforations, control of contamination process is essential for successful treatment [2]. Before the perforation sealing using MTA, a calcium hydroxide paste can be used to disinfect the perforation area and prevent granulation tissue invagination [23]. In the present cases, a calcium hydroxide intracanal medication was used to fill the root canals and perforation cavities. When combined with different vehicles, calcium hydroxide provides a strong base that can stimulate the mineralization process and decontaminate the surrounding environment [15, 24]. However, calcium hydroxide is a soluble material in the presence of tissue fluids [24]. In the treatment of perforations, calcium hydroxide does not result in the formation of a mineralized tissue barrier [25].

The use of a biological material is required to seal a perforation cavity. MTA is hygroscopic, promotes expansion, and seals the perforation cavity [26]. During the hydration process, the calcium silicates react to form a calcium hydroxide and hydrous silicate gel with a high alkaline pH [11, 23]. Furthermore, MTA is a biocompatible substrate that provides cell adhesion and differentiation stimulating the mineralized tissue formation [6]. It is considered to be a nonirritating bioactive silicate cement that is capable of stimulating the biosynthesis activity of the periodontal ligament cells and to play a role in cement formation and induction of bone tissue repair [23, 27]. In MTA-hydrated cement, calcium hydroxide sedimentation is lower than in Portland cement [11]. By studying the cytomorphology of osteosarcoma cells, it has been proved that Portland cement is a nonirritant material that does not affect the structural integrity of cells [28].

The biological evaluation of Mineral Trioxide Aggregate, Portland cement, or calcium hydroxide showed that the mechanisms of action of the materials are similar [29]. Also, the behavior of dog dental pulp with MTA or Portland Cement showed similar comparative results when used in direct pulp protection after pulpotomy [17]. Therefore, satisfactory results with the use of PC in pulpotomies [18, 19] and to induce apical healing have been demonstrated in teeth with open apices [16]. Moreover, it has also been shown that PC contains the basic elemental composition of MTA, except the presence of bismuth oxide [15] and considerable levels of calcium oxide which has an important role on tissue biological response from its conversion into calcium hydroxide and, consequently, stimulating tissue mineralization [30].

## 4. Conclusion

The positive clinical outcomes of these presented cases permit the new opportunity to discuss the use of CP as sealing material in root canal perforation.
